# Ammonium-nitrate mixtures dominated by NH_4_^+^-N promote the growth of pecan (*Carya illinoinensis*) through enhanced N uptake and assimilation

**DOI:** 10.3389/fpls.2023.1186818

**Published:** 2023-05-29

**Authors:** Mengyun Chen, Kaikai Zhu, Junyi Xie, Junping Liu, Zhenbing Qiao, Pengpeng Tan, Fangren Peng

**Affiliations:** ^1^ College of Forestry, Nanjing Forestry University, Nanjing, China; ^2^ Co-Innovation Center for Sustainable Forestry in Southern China, College of Forestry, Nanjing Forestry University, Nanjing, China; ^3^ Department of Ecology, Nanjing Forestry University, Nanjing, China

**Keywords:** pecan, nitrogen uptake and assimilation, NH_4_^+^–N, NO_3_^–^–N, gene expression

## Abstract

Nitrogen (N) limits plant productivity, and its uptake and assimilation may be regulated by N sources, N assimilating enzymes, and N assimilation genes. Mastering the regulatory mechanisms of N uptake and assimilation is a key way to improve plant nitrogen use efficiency (NUE). However, it is poorly known how these factors interact to influence the growth process of pecans. In this study, the growth, nutrient uptake and N assimilation characteristics of pecan were analyzed by aeroponic cultivation at varying ${\text{NH}}_{4}^{\;+}$ / ${\text{NO}}_{3}^{\;-}$ ratios (0/0, 0/100,25/75, 50/50, 75/25,100/0 as CK, T1, T2, T3, T4, and T5). The results showed that T4 and T5 treatments optimally promoted the growth, nutrient uptake and N assimilating enzyme activities of pecan, which significantly increased aboveground biomass, average relative growth rate (RGR), root area, root activity, free amino acid (FAA) and total organic carbon (TOC) concentrations, nitrate reductase (NR), nitrite reductase (NiR), glutamine synthetase (GS), glutamate synthase (Fd-GOGAT and NADH-GOGAT), and glutamate dehydrogenase (GDH) activities. According to the qRT-PCR results, most of the N assimilation genes were expressed at higher levels in leaves and were mainly significantly up-regulated under T1 and T4 treatments. Correlation analysis showed that a correlation between N assimilating enzymes and N assimilating genes did not necessarily exist. The results of partial least squares path model (PLS-PM) analysis indicated that N assimilation genes could affect the growth of pecan by regulating N assimilation enzymes and nutrients. In summary, we suggested that the ${\text{NH}}_{4}^{\;+}$ / ${\text{NO}}_{3}^{\;-}$ ratio of 75:25 was more beneficial to improve the growth and NUE of pecan. Meanwhile, we believe that the determination of plant N assimilation capacity should be the result of a comprehensive analysis of N concentration, N assimilation enzymes and related genes.

## 1 Introduction

Nitrogen (N) is the most important mineral nutrient required for plant growth and development (Guo et al., 2019). most nonlegume plants require their roots to take up 20-50 g N for every 1 kg of dry biomass produced (Xu et al., 2012). N fertilizer was the most widely used fertilizer in the world, and the suitable amount of N fertilizer can help improve crop yield and quality (Lu et al., 2021). However, the utilization efficiency of N fertilizer was very poor, with only about 30% (Guo et al., 2020). The loss of large amounts of N fertilizer has caused adverse effects such as land acidification, water pollution and greenhouse gas emissions (Qin et al., 2021). Therefore, improving N uptake and utilization by plants is important to promote better plant growth using less N fertilizer.

${\text{NH}}_{4}^{\;+}$ and ${\text{NO}}_{3}^{\;-}$ are the two main forms of N absorbed by plants, and studies have shown that the mixture of ${\text{NH}}_{4}^{\;+}$ and ${\text{NO}}_{3}^{\;-}$ is more beneficial to promote plant growth and N uptake. The maximum biomass of C. *paliurus* (*Cyclocarya paliurus*) was reached when the ${\text{NH}}_{4}^{\;+}$ : ${\text{NO}}_{3}^{\;-}$ ratio was 50:50 (Qin et al., 2021, 2). And the mixed application of ${\text{NH}}_{4}^{\;+}$ and ${\text{NO}}_{3}^{\;-}$ also increased the biomass, leaf area and TN concentration of black walnut (*Juglans nigra* L.) (Nicodemus et al., 2008). Zhang et al. (2007) showed that P, K^+^, Ca^2+^, and Mg^2+^ concentrations significantly varied under different N forms, while N forms affected the uptake of various mineral nutrients by plants in three main ways. Firstly, the N form can affect the absorption of mineral elements by regulating the balance of anions and cations (Egenolf et al., 2021), with antagonistic effects between ${\text{NH}}_{4}^{\;+}$ and cations and between ${\text{NO}}_{3}^{\;-}$ and anions (M’rah Helali et al., 2010). Second, the intake of ${\text{NH}}_{4}^{\;+}$ and ${\text{NO}}_{3}^{\;-}$ by plants will change the pH of the medium, the absorption of ${\text{NH}}_{4}^{\;+}$ will acidify the medium, the absorption of ${\text{NO}}_{3}^{\;-}$ will alkalize the medium, and the pH will directly affect the absorption of mineral elements (Zhao and Shen, 2018). Third, the uptake of ${\text{NO}}_{3}^{\;-}$ by plants requires higher energy cost than the uptake of ${\text{NH}}_{4}^{\;+}$ , which leads to slower absorption of other mineral elements (Tang et al., 2020).

Once ${\text{NH}}_{4}^{\;+}$ and ${\text{NO}}_{3}^{\;-}$ enter the plant through root uptake, most ${\text{NH}}_{4}^{\;+}$ will be assimilated in the roots, while ${\text{NO}}_{3}^{\;-}$ will be mainly assimilated in the leaves (Ruan et al., 2016). ${\text{NO}}_{3}^{\;-}$ can be reduced to ${\text{NO}}_{2}^{\;-}$ in the cytoplasm by nitrate reductase (NR), which is the rate-limiting and key enzyme of ${\text{NO}}_{3}^{\;-}$ metabolism and is also involved in energy metabolism, water stress and photorespiration of plants (Polcyn and Garnczarska, 2009; Bloom, 2015). Subsequently, ${\text{NO}}_{2}^{\;-}$ forms ${\text{NH}}_{4}^{\;+}$ catalyzed by nitrite reductase (NiR). NR-related genes have two types, *Nia1*, which is specifically induced to be expressed by NADH, and *Nia2*, whose expression is based on NAD(P)H as a substrate (Harris et al., 2000). It was shown that deletion mutations in *AtNIA2* resulted in 90% reduction of NR activity in leaves, but *AtNIA1* and *AtNIA2* genes were overexpressed in the roots of NR-deficient mutants (Loqué et al., 2003). Loppes et al. (1999) found that *Nia* expression was down-regulated by the presence of ${\text{NH}}_{4}^{\;+}$ , while ${\text{NO}}_{3}^{\;-}$ and ${\text{NO}}_{2}^{\;-}$ significantly up-regulated the expression of *Nia*. NiRs encoding genes include *nirS* and *nirK*, which encode heme *c* and heme *d_1_* (*cd_1_*-Nir) and copper (Cu-Nir), respectively. *nirS* is more widely distributed than *nirK* (Priemé et al., 2002). At present, little research has been reported on the response of NiR to N morphology.

Glutamine synthetase (GS) is a key enzyme for N assimilation and reactivation, and it forms the GS-GOGAT cycle with glutamate synthase (GOGAT) (Li et al., 2017). ${\text{NH}}_{4}^{\;+}$ synthesizes a Glutamine (Gln) *via* GS with a glutamate (Glu), while Glu is synthesized by the action of Gln and two GOGAT. Meanwhile, glutamate dehydrogenase (GDH) located in mitochondria can also synthesize Glu directly using 2-ketoglutarate and ${\text{NH}}_{4}^{\;+}$ (Krapp, 2015). GS includes two isoforms, cytosolic GS1 and plastidic GS2, GS1 is the main enzyme in non-photosynthetic tissues or roots which is responsible for primary ${\text{NH}}_{4}^{\;+}$ assimilation in roots or for re-assimilation of ${\text{NH}}_{4}^{\;+}$ produced in leaves during protein turnover, while GS2 is mainly responsible for the assimilation of ${\text{NH}}_{4}^{\;+}$ produced by photorespiration in chloroplasts (Thomsen et al., 2014). GS-related genes in plants have two types, one is *Gln1* located in the cytoplasm and the other is *Gln2* in the chloroplast (Bernhard and Matile, 1994). *Gln1* was not induced by light and was mainly expressed in plant roots, while *Gln2* expression was induced by light and was majorly present in the aboveground of plants (Edwards and Coruzzi, 1989). In *Arabidopsis thaliana*, *GLN1;2* was significantly up-regulated in response to ${\text{NH}}_{4}^{\;+}$ induction (Guo et al., 2004), whereas *gln1;2* mutants exhibited lower GS activity and higher ${\text{NH}}_{4}^{\;+}$ concentrations in the presence of adequate ${\text{NO}}_{3}^{\;-}$ supply (Lothier et al., 2011). Based on the electron donor, GOGAT can be divided into two types: ferredoxin-dependent (Fd-GOGAT) and NADH-dependent (NADH-GOGAT) enzymes (Li et al., 2017). *GLU1* and *GLU2* are two Fd-GOGATs identified in *Arabidopsis*, while *GLU1* plays a main role in photorespiration and N assimilation in leaves, *GLU2* may play a major role in primary N assimilation in roots (Coschigano et al., 1998). *GLT* is the only *NADH-GOGAT* gene in *Arabidopsis* that functions in non-photosynthetic ${\text{NH}}_{4}^{\;+}$ assimilation and Glu synthesis (Lancien et al., 2002).

The pecan [*Carya illnoinensis* (Wangenh.) K. Koch] is a member of the Juglandaceae family. Pecans have thin shells and full, sweet kernels, which makes them a world-renowned economic tree (Zhu et al., 2020). However, pecans have the same problem of low nitrogen use efficiency (NUE) in cultivation, and hardly any relevant reports are available. Therefore, in this experiment, pecan seedlings were used as materials to study the effects of different ${\text{NH}}_{4}^{\;+}$ : ${\text{NO}}_{3}^{\;-}$ ratios on their growth and development and N assimilation. Specifically, biomass, leaf area, root growth, mineral element content, N uptake, N assimilation enzymes, and expression of related genes were measured to address the following questions: (A) Which ${\text{NH}}_{4}^{\;+}$ : ${\text{NO}}_{3}^{\;-}$ ratio is more helpful to enhance the growth and NUE of pecan seedlings under certain N concentration? (B) How N assimilation genes, N assimilation enzymes, and N interact to affect the growth and development of pecan?

## 2 Material and methods

### 2.1 Plant material and experimental design

This experiment was conducted in the underground greenhouse of Nanjing Forestry University from April 18, 2021 to June 9, 2021. The seedlings of pecan “Pawnee” seeds were used as experimental materials. The seedlings with a height of about 25 cm were selected, and the roots were cleaned and disinfected for indoor aeroponic cultivation trials. Each treatment had 18 seedlings, arranged according to a randomized complete block design. Greenhouse conditions were as follows: natural light, 12h/12h day/night, day and night temperature of 30/25°C, relative humidity of 70% ± 5%. The nutrient solution was modified Hoagland nutrient solution. The formula was as follows: 1.25 mM Ca(NO_3_)_2_, 0.5 mM Ca(H_2_PO_4_)_2_, 1.0 mM K_2_SO_4_, 0.5 mM MgSO_4_, 1.0 μM ZnSO_4_, 12.5 μM H_3_BO_3_, 1.0 μM MnSO_4_, 0.25 μM CuSO_4_, 0.1 μM (NH_4_)_6_Mo_7_O_24_, 10 μM EDTA-Fe. The pH was adjusted to approximately 6.0 every other day with 24 h aeration, and the nutrient solution was replaced every 7 days. The seedlings were precultured with 1/4 nutrient solution for one week, and then cultured in total nutrient solution for experimental treatment. The nitrogen concentration in the nutrient solution was 2 mM. Based on the same N supply, the five ammonium-nitrate ratios ( ${\text{NH}}_{4}^{\;+}$ : ${\text{NO}}_{3}^{\;-}$ ) were 0:100, 25:75, 50:50, 75:25, and 100:0, corresponding to T1, T2, T3, T4, and T5, respectively. The nutrient solution without N was used as the control (CK), and each treatment was repeated 3 times, each with 6 seedlings. (NH_4_)_6_Mo_7_O_24_ was replaced by (Na)_6_Mo_7_O_24_, (NH_4_)_2_SO_4_ was used as the ammonium source, and Ca(NO_3_)_2_ was used as the nitrate source. Samples were taken after 45 days of treatment for further determination.

### 2.2 Measurements

#### 2.2.1 Growth parameters

To evaluate the effects of different N forms on the growth of pecans, pecan seedlings cultivated for 45 d under different treatments were used for the determination of growth indicators. Aboveground and underground biomass (g), which were measured by weighing. Leaf area (mm^2^), which was measured by weighing method and LI-3000 leaf areometer. Pecan roots were scanned with a digital scanner (STDl600EpsonUSA) and root length, root surface area, and root volume were determined with winRhizo root analysis software. Root activity (mg g^-1^ h^-1^) was determined by TTC redox method (Stūrīte et al., 2005). And the average relative growth rate (RGR) (g g^-1^ d^-1^) calculation formula is as follows:


RGR=(lnW2−lnW1)/Δt

Where W1 and W2 represent the total pecan biomass (g) of the same plant before and after treatment, respectively. Δt denotes the time interval (d) between the two measurements.

#### 2.2.2 Biochemical traits

To evaluate the effects of different N forms on the biochemical traits of pecan seedlings, Total nitrogen (TN), total phosphorus (TP), and total potassium (TK) concentrations, which were determined by HClO_4_-H_2_SO_4_ decoction method. The total organic carbon (TOC) concentrations were determined by K_2_Cr_2_O_7_ volumetric and external heating method. And free amino acid (FAA) concentration measurements refer to GB/T 8312-87 to GB/T 8314-87.

#### 2.2.3 Nitrogen assimilation

To evaluate the effect of different N forms on the nutrient absorption of pecans, the nitrate reductase (NR), nitrite reductase (NiR), glutamine synthetase (GS), glutamate synthase (Fd-GOGAT and NADH-GOGAT), and glutamate dehydrogenase (GDH) activity in roots and leaves were determined by enzyme activity kit (Jiancheng, Nanjing, China). NR catalyzes the reduction of ${\text{NO}}_{3}^{\;-}$ to ${\text{NO}}_{2}^{\;-}$ by NADH, and the NR activity was expressed by measuring the NADH reduction rate. NiR reduces ${\text{NO}}_{2}^{\;-}$ to NO, and the NiR activity was calculated according to the reduction rate of ${\text{NO}}_{2}^{\;-}$ . In the presence of ATP and Mg^2+^, GS catalyzes ${\text{NH}}_{4}^{\;+}$ and glutamic acid to synthesize glutamine, which is further converted into γ-glutamyl hydroxamic acid. The complex formed under acidic conditions was measured to calculate GS activity. Fd-GOGAT catalyzes the transfer of the amino group of glutamine to α-ketoglutarate and forms glutamic acid. The dehydrogenation of glutamic acid produces NADH, which makes WST-8 orange-yellow. The absorbance value was measured to indicate Fd-GOGAT activity. NADH-GOGAT catalyzes the transfer of the amino group of glutamine to α-ketoglutarate to form glutamic acid, while NADH is oxidized to NAD^+^. The NADH-GOGAT activity was reflected by measuring the NADH decline rate. GDH catalyzes ${\text{NH}}_{4}^{\;+}$ , α-ketoglutarate and NADH to produce glutamic acid and NAD^+^, and GDH activity was expressed by measuring the rate of NADH reduction.

#### 2.2.4 Expression analysis

According to the manufacturer’s protocol, the total RNA was extracted from the leaves and roots of pecan using a Universal Plant Total RNA Extraction Kit (Bioteke, Beijing, China) and stored at -80°C until further use. The purity and integrity of the isolated total RNA was analyzed by agarose gel electrophoresis and Nanodrop 2000 spectrophotometer (Thermo Scientific, Wilmington, NC, USA). First-strand cDNA was synthesized using a cDNA Synthesis Kit (HiScript ^®^RIII RT SuperMix for qPCR +gDNA wiper, Vazyme, Nanjing, China). The qRT-PCR was performed on a 7500 Real-Time PCR system (Applied BiosystemsTM, Foster City, CA, USA) using a Taq Pro Universal SYBR qPCR Master Mix (Vazyme, Nanjing, China). The specific primers were synthesized by Tsingke Biotechnology Ltd (Nanjing, China), the details of the primers were provided in **Table 1**, melting curve analyses were used to verify the efficiency of the primers (**Supplementary Figure S1**). The Actin gene was used as an internal reference gene (Zhu et al., 2022), and the relative expression levels of pecan Nia, NiR, Gln, GOGAT, and GDH genes were determined using the 2^-ΔΔCt^ method (Livak and Schmittgen, 2001). Values represent mean calculated from three biological replicates and three technological repeats. The pecan N assimilate genes were renamed according to the *Arabidopsis* gene name and the NCBI pblast results.

**Table 1:** The sequences of primers used for qPCR.

No.	Gene Name	Gene ID	Forward Primer Sequence(5´-3´)	Reverse Primer Sequence(5´-3´)
1	CiNia2a	CiPaw.05G228100	ACGCGTATTCGGTATTGCAG	CACGAGTCACTTTCCTCCCC
2	CiNia2b	CiPaw.06G025400	AAACTCGTGGACAACGCAGA	CACTTTTCTCCCACCTCCAGAA
3	CiNiR1a	CiPaw.05G006800	GGTCCAAGCAGACGACATGA	GCTCCACAATGAGCTGGAGT
4	CiNiR1b	CiPaw.06G172200	CCCAAAAGCAGCTGGAGAGA	TGCTTCTGTGGATGGACACC
5	CiGln2a	CiPaw.01G157900	CATCCGCCATTCCTGATCTGA	CCCCACATCTTTGCTGTCGT
6	CiGln2b	CiPaw.02G090300	TTGTAAGGGCTTCCCCCACC	CTGTGCCATTTTCACCTCGG
7	CiGln1.2	CiPaw.16G097000	CGCTAAAATCGCCTGTTGGG	ACCCGATCCACCGATCCATA
8	CiFd-GOGATa	CiPaw.09G047200	TGTGGCGTCGTGGAATGTAA	TGGCGTCTACGACCTTTCAC
9	CiFd-GOGATb	CiPaw.10G039900	GACGTCTTCGGCCGTCAA	CCAAGTTTGCAACCCTCGGTC
10	CiNADH-GOGAT	CiPaw.05G252100	CTCTGTCCGCAAGGACTGTT	TCAGGGCCATGTAGCTCGTA
11	CiGDHa	CiPaw.02G142100	AAGGAGTGTCCTGAAGCTTTG	GCACGGGCATTATTGTAAGAG
12	CiGDHb	CiPaw.01G227200	CTCAACGACCAGACGGAGAC	ACCTGCAGCACTCAACTCAAT
13	Actin	CiPaw.03G124400	ACAGGGAGAAGATGACTCAAATC	CACTGGCATACAGAGAGAGAAC

### 2.3 Data analysis

Before analysis of variance (ANOVA), data were checked for normality and homogeneity of variances. One-way ANOVA was performed to test the effects of different N forms on biomass, leaf area, root growth, N uptake rate, and N transport capacity of pecan. Two-way ANOVA was performed to test the effects of N form, organ and their interactions on the nutrients, enzyme activity and gene expression of pecan. Differences were considered significant at *p*<0.05. Correlation analysis was used to test the correlations between the growth and N assimilation indicators. Partial least squares path model (PLS-PM) was used to analyze the role between N assimilation genes, N assimilation enzymes, plant nutrients and growth. Finally, principal component analysis (PCA) was carried out on 20 indicators of N assimilation, determining the number of principal components according to characteristic values and cumulative contribution rates and calculating principal component scores based on factor scores (Rahmani and Atia, 2017).

All statistical analyses were performed with SPSS 23.0 software (Version 23.0, Chicago, IL, USA) and r4.2.1, the partial least squares path model was constructed using the “plspm” package (Latan and Noonan, 2017). All charts were drawn with r4.2.1 and SigmaPlot (Version 14.0, Barcelona, Spain).

## 3 Results

### 3.1 Growth analysis of pecan under different N forms

**Figure 1:**
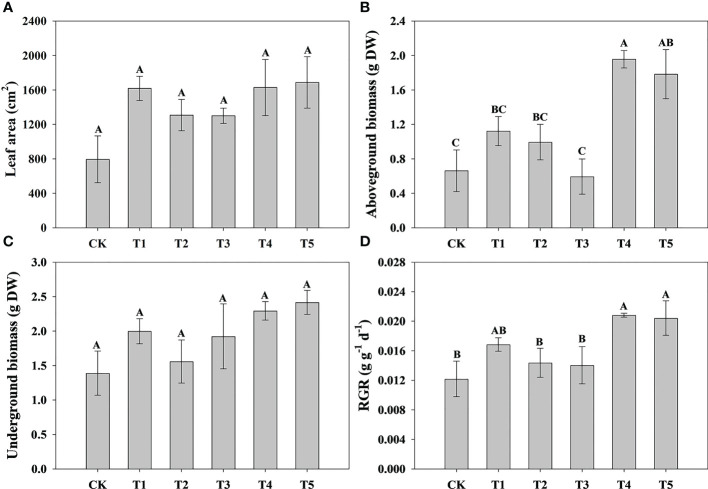
Analysis of leaf area and biomass of pecan under different N forms. Leaf area of pecan under different N forms **(A)**. Aboveground biomass of pecan under different N forms **(B)**. Underground biomass of pecan under different N forms **(C)**. RGR of pecan under different N forms **(D)**. Uppercase letters indicate differences between N form treatments, at *p<* 0.05. RGR, average relative growth rate; DW, dry weight.

**Figure 2:**
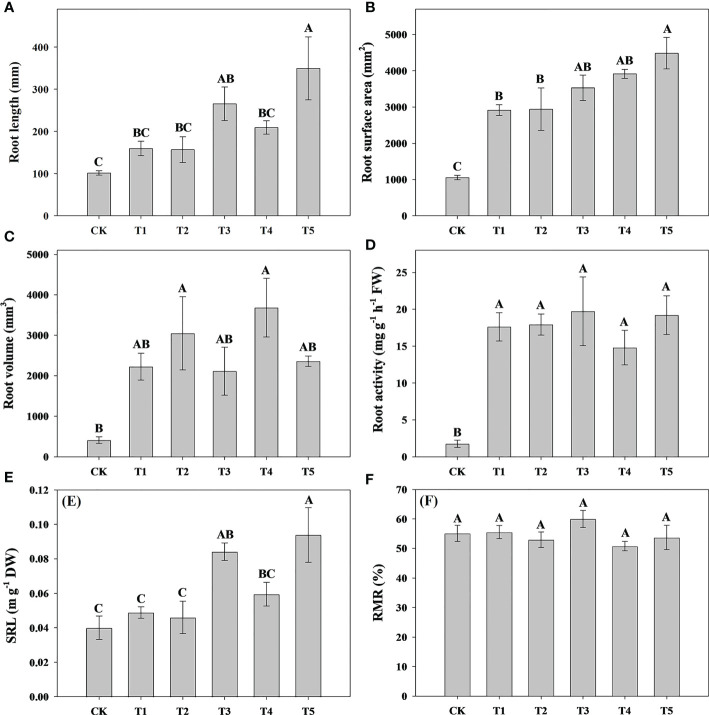
Analysis of root length, root surface area, root volume, and root activity of pecan under different N forms. Root length of pecan under different N forms **(A)**. Root surface area of pecan under different N forms **(B)**. Root volume of pecan under different N forms **(C)**. Root activity of pecan under different N forms **(D)**. SRL of pecan under different N forms **(E)**, SRL is the ratio of root length to root biomass. RMR of pecan under different N forms **(F)**, RMR is the ratio of root biomass to whole plant biomass. Uppercase letters indicate differences between N form treatments, at *p<* 0.05. SRL, specific root length; RMR, root mass ratio; FW, fresh weight; DW, dry weight.

To explore the effect of different N forms on the growth characteristics of pecan, we analyzed pecan biomass, leaf area and root growth under different N forms (**Figure 1**, **2**). The results showed that the leaf area of pecan was not significantly affected by different N form treatments (**Figure 1A**). T4 and T5 significantly increased aboveground biomass of pecan (*p*< 0.05), other treatments were not significantly different from CK (**Figure 1B**). However, there was no significant effect of N form on underground biomass of pecan (**Figure 1C**). T4 and T5 were significantly increased the RGR of pecan (*p*< 0.05), other treatments were not significantly different from CK (**Figure 1D**). T4 and T5 significantly increased the root length (*p*< 0.05), while the other treatments were not significantly different from CK (**Figure 2A**). Both T1-T5 treatments significantly increased root area and root activity compared to CK (*p*< 0.05) (**Figures 2B, D**). T2 and T4 significantly increased the root volume (*p*< 0.05), while the other treatments were not significantly different from CK (**Figure 2C**). T3 and T5 treatments significantly increased SRL (*p*< 0.05), and T1, T2, and T4 treatments were not significantly different from CK (**Figure 2E**). Different N forms had no significant effect on RMR of pecan (**Figure 2F**).

### 3.2 Analysis of N uptake and transport of pecan under different N forms

**Figure 3:**
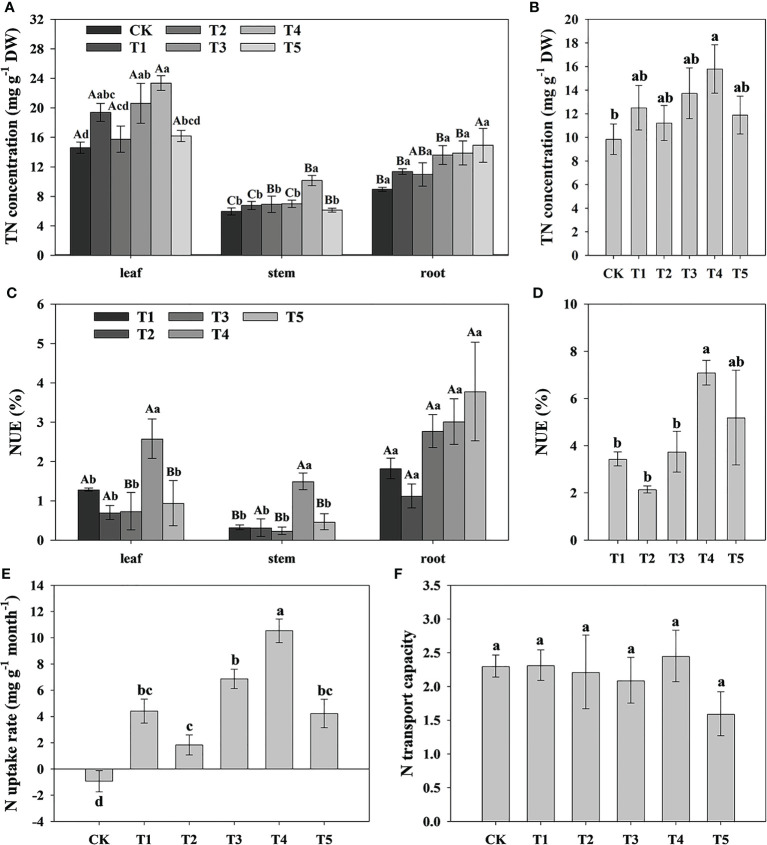
Analysis of N uptake rate and N transport capacity of pecan under different N forms. TN concentrations in leaves, stems and roots of pecan under different N forms **(A)**. The mean level of TN concentrations of pecan leaves, stems and roots under different N forms **(B)**. NUE of leaves, stems and roots of pecan under different N forms **(C)**, NUE was measured by the ratio of the difference between the TN accumulation of the N application treatment and the TN accumulation of the CK treatment to the total N application. The sum of NUE of pecan leaves, stems and roots under different N forms **(D)**. N uptake rate of pecan under different N forms **(E)**, N uptake rate was measured by the ratio of the differential of TN concentration before and after fertilization to the time. N transport capacity of pecan under different N forms **(F)**, N transport capacity was measured by the ratio of TN concentration in the aboveground and underground of the plant. Uppercase letters indicate differences between organs, and lowercase letters indicate differences between ${\text{NH}}_{4}^{\;+}$ : ${\text{NO}}_{3}^{\;-}$ treatments, at *p<* 0.05. TN, Total nitrogen; NUE, nitrogen use efficiency; DW, dry weight.

To study the effect of different N forms on the N uptake and assimilation of pecan, we analyzed the TN concentration in all organs of pecan under different N forms (**Table 2**, **Figure 3**). According to **Table 2**, we found that both N form and plant organ had a highly significant effect on TN (*p*< 0.01), but the interaction between them had no significant effect on TN. The TN concentrations in pecan leaves were greater than that in the stems and roots (**Figure 3A**). In leaves, T1, T3, and T4 treatments were not significantly different but significantly greater than CK (*p*< 0.05), and T2 and T5 were not significantly different from CK (**Figure 3A**). In the stems, the T4 was significantly higher than the other treatments (*p*< 0.05), and there was no significant difference between other treatments (**Figure 3A**). In the roots, the difference between treatments was not significant (**Figure 3A**). According to the mean value of pecan leaf, stem, and root, the T4 was significantly higher than CK (*p*< 0.05), and they were not significantly different from the other treatments (**Figure 3B**). T4 treatment significantly increased the NUE of leaves and stems (*p*< 0.05), and the average NUE of leaves, stems, and roots also showed that T4 treatment was significantly higher than T1, T2, and T3 treatments (*p*< 0.05) (**Figures 3C, D**). Both N form treatments were significantly increased N uptake rate of pecan (*p*< 0.05), with the most significant being the T4 treatment (**Figure 3E**). surprisingly, the N uptake rate of pecan under CK was negative, indicating that the N efflux from pecan was greater than the influx under CK (**Figure 3E**). Both N forms and CK treatment had no significant effect on N transport capacity of pecan (**Figure 3F**).

**Table 2:** ANOVAs on the effects of N form and organ on FAA and nutrient elements in pecan.

	TN		FAA		TP		TK		TOC	
Effects	F	*p*	F	*p*	F	*p*	F	*p*	F	*p*
N form	4.122	**< 0.01**	3.122	**0.026**	2.664	**0.047**	5.852	**< 0.01**	0.648	0.665
Organ	61.638	**< 0.01**	42.687	**< 0.01**	0.036	0.965	0.942	0.404	1.858	0.178
N form and Organ	1.478	0.208	1.583	0.172	2.090	0.068	0.923	0.529	0.463	0.897

The correlation reached a significant level (p< 0.05), and the correlation was extremely significant (p< 0.01). Significant results are shown in bold. TN, Total nitrogen; FAA, Free amino acid; TP, Total phosphorus; TK, Total potassium; TOC, Total organic carbon.

### 3.3 Analysis of FAA, TP, TK, and TOC concentration of pecan under different N forms

**Figure 4:**
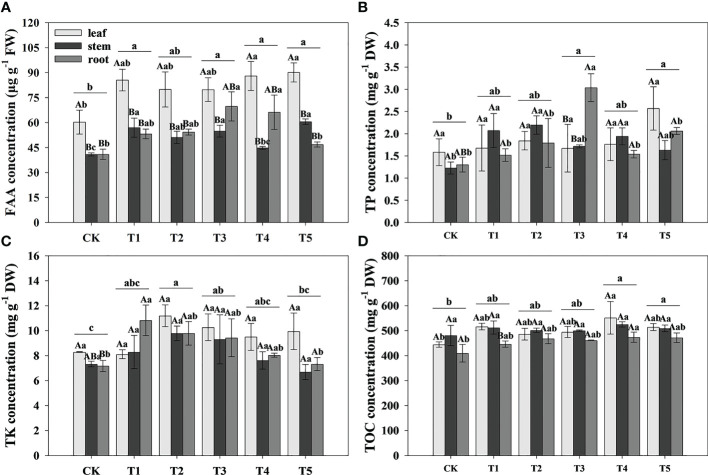
Analysis of FAA and nutrient elements concentration of pecan under different N forms. FAA concentrations in leaves, stems and roots of pecan under different N forms **(A)**. TP concentrations in leaves, stems and roots of pecan under different N forms **(B)**. TK concentrations in leaves, stems and roots of pecan under different N forms **(C)**. SOC concentrations in leaves, stems and roots of pecan under different N forms **(D)**. Uppercase letters indicate differences between organs, and lowercase letters indicate differences between ${\text{NH}}_{4}^{\;+}$ : ${\text{NO}}_{3}^{\;-}$ treatments, at *p<* 0.05. FAA, Free amino acid; TP, Total phosphorus; TK, Total potassium; TOC, Total organic carbon; FW, fresh weight; DW, dry weight.

To study the effect of different N forms on the FAA and nutrient elements of pecan, we analyzed the FAA, TP, TK, and TOC concentration in all organs of pecan under different N forms (**Table 2**, **Figure 4**). We found that N form had a highly significant effect on TK (*p*< 0.01) and a significant effect on FAA and TP (*p*< 0.05), plant organ had a strongly significant effect on FAA (*p*< 0.01), neither N form nor plant organ had a significant effect on TOC, and the interaction between them had no significant effect on FAA and all nutrient elements (**Table 2**). We found that FAA concentrations were highest in the leaves of pecan (**Figure 4A**). In the leaves, the FAA concentrations of pecan under T4 and T5 were significantly greater than CK (*p*< 0.05), and T1, T2, and T3 were not significantly different from other treatments (**Figure 4A**). In the stems, the FAA concentrations of pecan under T1, T3, and T5 were significantly greater than CK and T4 (*p*< 0.05), and T2 was significantly greater than CK (*p*< 0.05) and not significantly different from the other treatments (**Figure 4A**); The TP concentrations under T1, T2, and T4 were significantly greater than CK (*p*< 0.05), and T3 and T5 were not significantly different from the other treatments (**Figure 4B**). In the roots, the FAA concentrations were significantly greater under T3 and T4 than CK and T5 (*p*< 0.05), and T1 and T2 were not significantly different from the other treatments(**Figure 4A**); The TP concentrations under T4 were significantly greater than the other treatments (*p*< 0.05) (**Figure 4B**); The TK concentrations under T1 were significantly greater than CK and T5 (*p*< 0.05), and T2, T3, and T4 were not significantly different from the other treatments (**Figure 4C**); The TOC concentrations under T4 were significantly greater than CK (*p*< 0.05), and T1, T2, T3, and T5 were not significantly different from the other treatments (**Figure 4D**). According to the mean value of pecan leaf, stem, and root, the FAA concentrations of pecan under T1, T3, T4, and T5 were significantly greater than CK (*p*< 0.05), and T2 was not significantly different from the other treatments (**Figure 4A**); the TP concentrations of pecan under T3 and T5 were significantly greater than CK (*p*< 0.05), and T1, T2, and T4 were not significantly different from the other treatments (**Figure 4B**); the TK concentrations of pecan under T2 and T3 were significantly greater than CK (*p*< 0.05), and T1 and T4 were not significantly different from the other treatments (**Figure 4C**); the TOC concentrations of pecan under T4 and T5 were significantly greater than CK (*p*< 0.05), and T1, T2, and T3 were not significantly different from the other treatments (**Figure 4D**).

### 3.4 Analysis of N assimilation-related enzyme activity of pecan under different N forms

**Figure 5:**
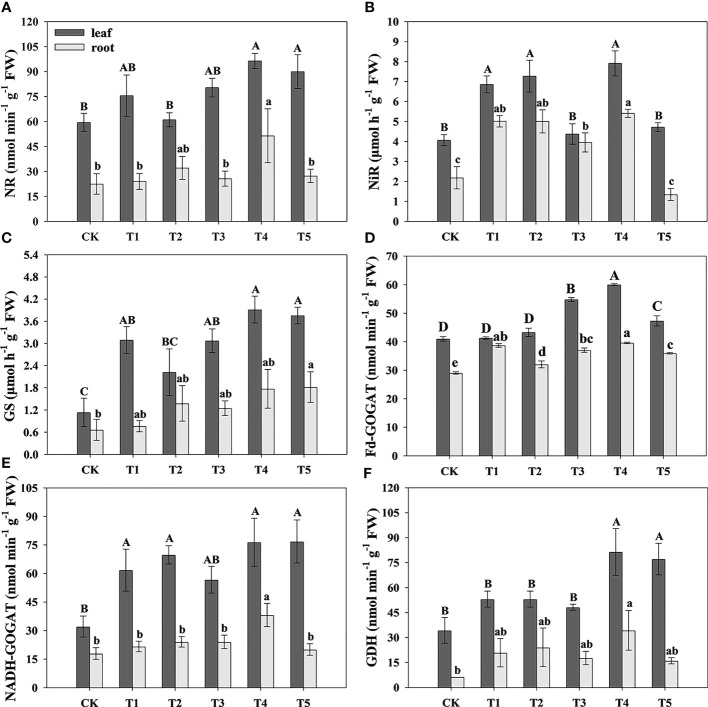
Analysis of N assimilation-related enzymes activity of pecan under different N forms. NR activity in leaves and roots of pecan under different N forms **(A)**. NiR activity in leaves and roots of pecan under different N forms **(B)**. GS activity in leaves and roots of pecan under different N forms **(C)**. GOGAT activity in leaves and roots of pecan under different N forms **(D)**. GDH activity in leaves and roots of pecan under different N forms **(E)**. Uppercase letters indicate differences between organs, and lowercase letters indicate differences between ${\text{NH}}_{4}^{\;+}$ : ${\text{NO}}_{3}^{\;-}$ treatments, at *p<* 0.05. NR, Nitrate reductase; NiR, Nitrite reductase; GS, Glutamine synthetase; GOGAT, Glutamate synthase; GDH, Glutamate dehydrogenase.

To research the effect of N form on N assimilation in pecan, we analyzed the N assimilation-related enzyme activity in leaves and roots of pecan under different N forms (**Figure 5**). In the leaves, the NR activity under T4 and T5 was significantly greater than CK and T2 (*p*< 0.05), and T1 and T3 were not significantly different from the other treatments (**Figure 5A**); the NiR activity under T1, T2, and T4 was significantly greater than CK and T5 (*p*< 0.05) (**Figure 5B**); the GS activity under all N form treatments was significantly greater than CK (*p*< 0.05) (**Figure 5C**); the F-GOGAT activity under T3, T4, and T5 was significantly greater than CK, T1, and T2 (*p*< 0.05), and CK, T1, and T2 treatments were not significantly different from each other, and T4 treatment was significantly greater than T3 and T5 (*p*< 0.05) (**Figure 5D**).The NADH-GOGAT activity under T1, T2, T4, and T5 was significantly greater than CK (*p*< 0.05), and T3 was not significantly different from the other treatments (**Figure 5E**); the GDH activity under T4 and T5 was significantly greater than CK, T1, T2, and T3 (*p*< 0.05) (**Figure 5F**). In the roots, the NR activity under T4 was significantly greater than CK, T1, T3, and T5 (*p*< 0.05), and T2 was not significantly different from the other treatments (**Figure 5A**); the NiR activity under T1, T2, T3, and T4 was significantly greater than CK and T5 (*p*< 0.05) (**Figure 5B**); the GS activity under T2, T4, and T5 was significantly greater than CK and T1 (*p*< 0.05), and CK, T1, and T3 were not significantly different from each other (**Figure 5C**); the Fd-GOGAT activity under T1, T3, and T4 treatments was significantly greater than CK, T2, and T5 (*p*< 0.05), and T1, T3, and T4 treatments were not significantly different from each other, and CK and T5 treatments had no significantly difference and less significantly than T2 (*p*< 0.05) (**Figure 5D**); the NADH-GOGAT activity under T4 was significantly greater than the other treatments (*p*< 0.05) (**Figure 5E**); the GDH activity under T1, T2, and T4 was significantly greater than CK (*p*< 0.05), and T3 and T5 were not significantly different from CK (**Figure 5F**).

### 3.5 Analysis of N assimilation genes expression of pecan under different N forms

**Figure 6:**
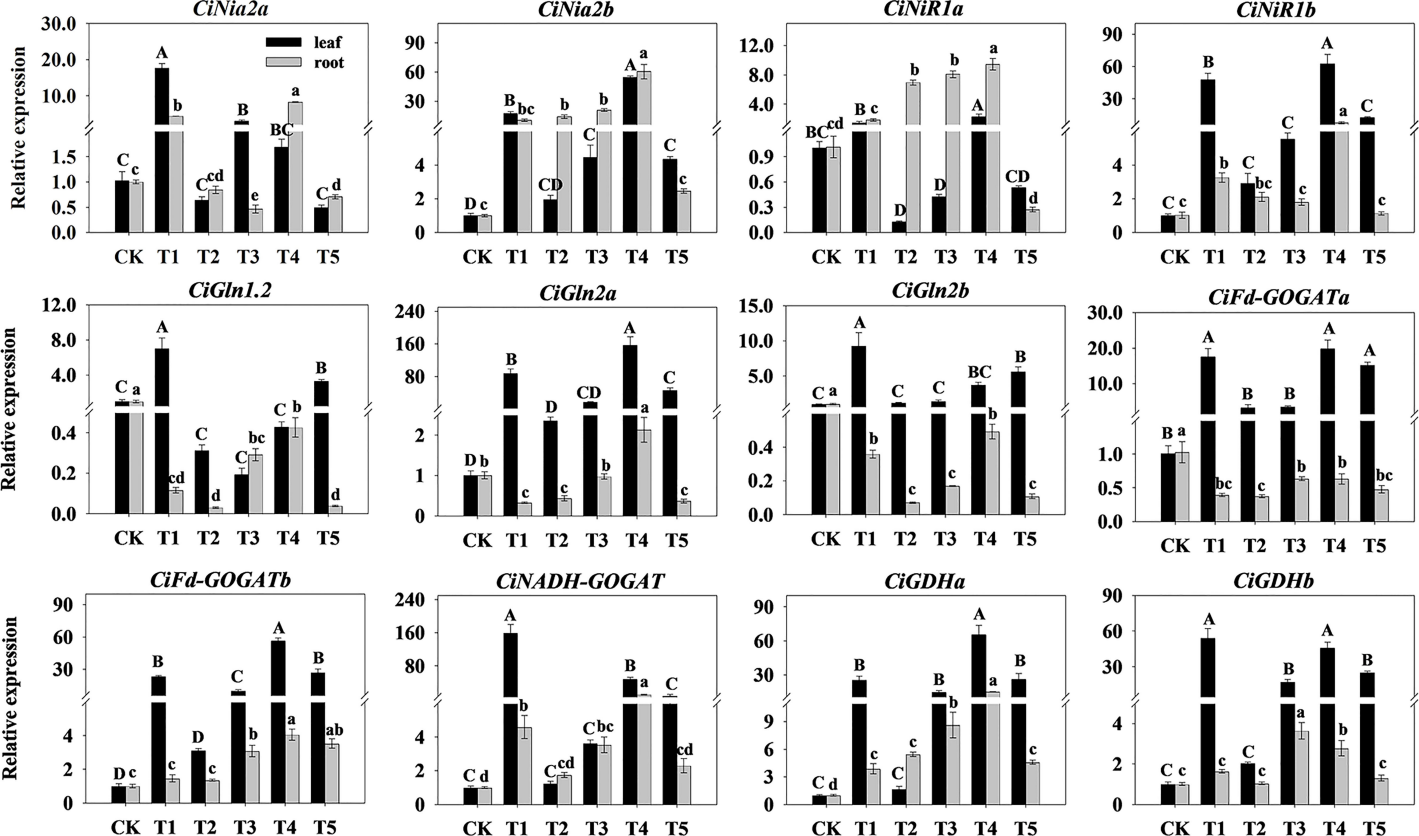
Relative expression of N assimilation-related enzymes genes in pecan under different N forms. The expression levels of *CiNia*, *CiNiR*, *CiGln*, *CiGOGAT*, and *CiGDH* genes in leaves and roots of pecan after varying ${\text{NH}}_{4}^{\;+}$ : ${\text{NO}}_{3}^{\;-}$ ratio treatments were quantified by qRT-PCR, with *Actin* as the reference gene. Different capital letters indicate significant differences in leaves (*p<* 0.05), and different lowercase letters indicate significant differences in roots (*p<* 0.05).

To further investigate the effect of N form on N assimilation in pecan, we analyzed the N assimilation enzyme-related genes in leaves and roots of pecan under different N forms (**Figure 6**). In the leaves, the expression levels of *CiNia2a*, *CiGln1.2*, *CiGln2b*, *CiFd-GOGATb*, and *CiNADH-GOGAT* under T1 were significantly up-regulated (*p*< 0.05); the expression levels of *CiNia2b*, *CiNiR1a*, *CiNiR1b*, *CiGln2a*, *CiFd-GOGATb*, and *CiGDHa* under T4 were significantly up-regulated (*p*< 0.05); the expression levels of *CiFd-GOGATa* under T1, T4, and T5 were significantly up-regulated (*p*< 0.05); and the expression levels of *CiGDHb* under T1 and T4 were significantly up-regulated (*p*< 0.05). In the roots, the expression levels of *CiNia2a*, *CiNia2b*, *CiNiR1a*, *CiNiR1b*, *CiGln2a*, *CiFd-GOGATb*, *CiNADH-GOGAT*, and *CiGDHa* under T4 were significantly up-regulated (*p*< 0.05); the expression levels of *CiGln1.2*, *CiGln2b*, and *CiFd-GOGATa* under CK were significantly up-regulated (*p*< 0.05); and the expression levels of *CiGDHb* under T1 and T3 were significantly up-regulated (*p*< 0.05).

### 3.6 Correlation analysis of N assimilation genes with growth and N assimilation enzymes in pecan

**Figure 7:**
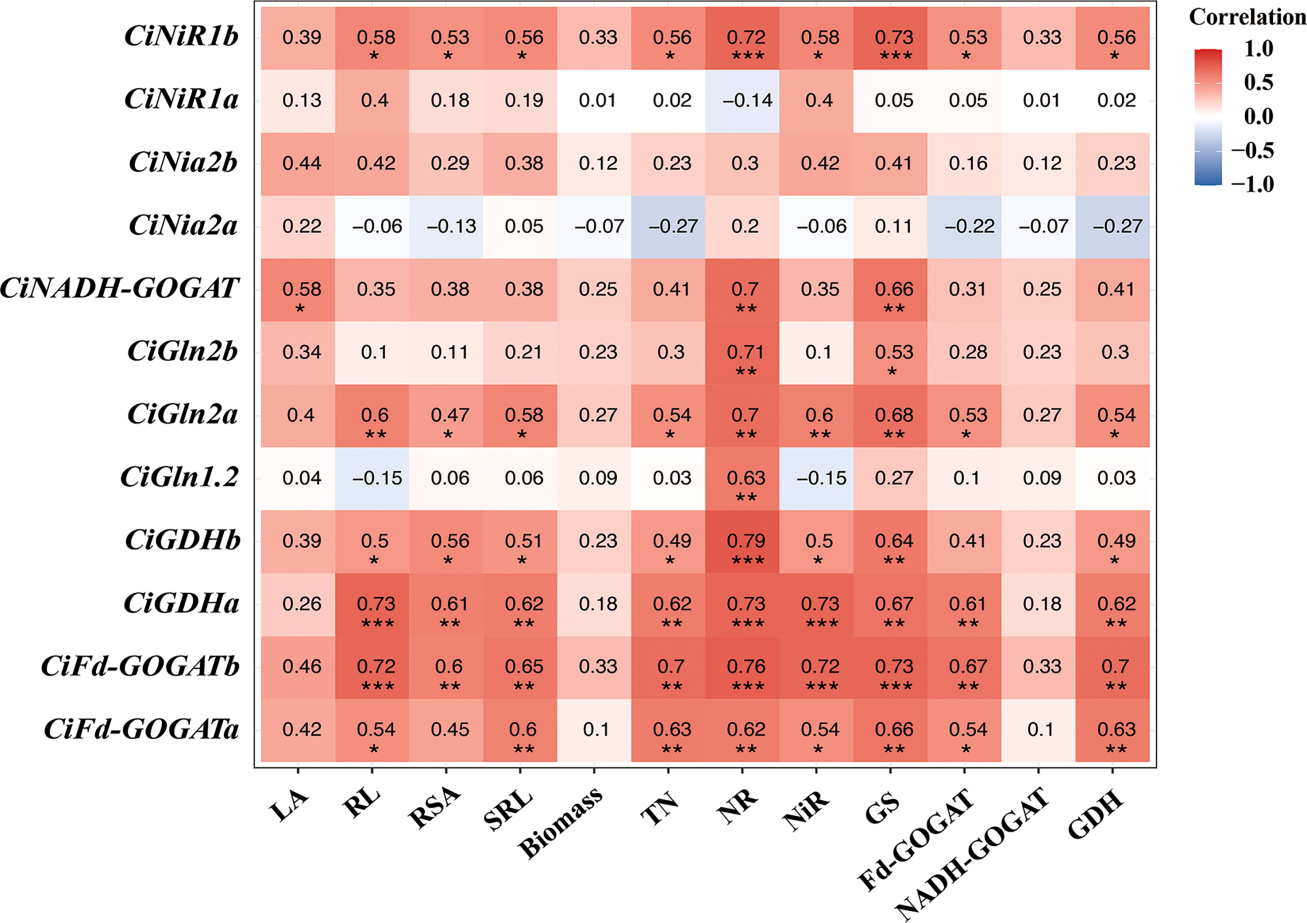
Correlation analysis of growth physiological indexes of pecan. *p< 0.05; **p< 0.01; ***p< 0.001. LA, Leaf area; RL, Root length; RSA, Root surface area; SRL, specific root length; TN, Total nitrogen; NR, Nitrate reductase; NiR, Nitrite reductase; GS, Glutamine synthetase; GOGAT, Glutamate synthase; GDH, Glutamate dehydrogenase.

Correlation analysis showed that most of the N assimilate enzyme genes were positively correlated with growth and N assimilate enzymes in pecan (**Figure 7**). Leaf area was significantly positively correlated with *CiNADH-GOGAT* (*p*< 0.05). Root length was significantly positively correlated with *CiNiR1b*, *CiFd-GOGATa*, and *CiGDHb* (*p*< 0.05), and highly significantly positively correlated with *CiGln2a*, *CiFd-GOGATb*, and *CiGDHa* (*p*< 0.01). Root surface area was significantly positively correlated with *CiNiR1b*, *CiGln2a*, and *CiGDHb* (*p*< 0.05), and highly significantly positively correlated with *CiGDHa* and *CiFd-GOGATb* (*p*< 0.01). SRL was significantly positively correlated with *CiNiR1b*, *CiGln2a*, and *CiGDHb* (*p*< 0.05), and highly significantly positively correlated with *CiFd-GOGATa*, *CiFd-GOGATb*, and *CiGDHa* (*p*< 0.01). TN was significantly positively correlated with *CiNiR1b*, *CiGln2a*, and *CiGDHb* (*p*< 0.05), and extremely significantly positively correlated with *CiFd-GOGATa*, *CiFd-GOGATb*, and *CiGDHa* (*p*< 0.01). NR was highly significantly positively correlated with *CiNiR1b*, *CiGln1.2*, *CiGln2a*, *CiGln2b*, *CiFd-GOGATa*, *CiFd-GOGATb*, *CiNADH-GOGAT*, *CiGDHa*, and *CiGDHb* (*p*< 0.01). NiR was significantly positively correlated with *CiNiR1b*, *CiFd-GOGATa*, and *CiGDHb* (*p*< 0.05), and extremely significantly positively correlated with *CiGln2a*, *CiFd-GOGATb*, and *CiGDHa* (*p*< 0.01). GS was significantly positively correlated with *CiGln2b* (*p*< 0.05), and extremely significantly positively correlated with *CiNiR1b*, *CiGln2a*, *CiFd-GOGATa*, *CiFd-GOGATb*, *CiNADH-GOGAT*, *CiGDHa*, and *CiGDHb* (*p*< 0.01). Fd-GOGAT was significantly positively correlated with *CiNiR1b*, C*iGln2a*, and *CiFd-GOGATa* (*p*< 0.05), and highly significantly positively correlated with *CiGDHa* and *CiFd-GOGATb* (*p*< 0.01). GDH was significantly positively correlated with *CiNiR1b*, *CiGln2a*, and *CiGDHb* (*p*< 0.05), and highly significantly positively correlated with *CiFd-GOGATa*, *CiFd-GOGATb*, and *CiGDHa* (*p*< 0.01).

### 3.7 The partial least squares path model analysis

**Figure 8:**
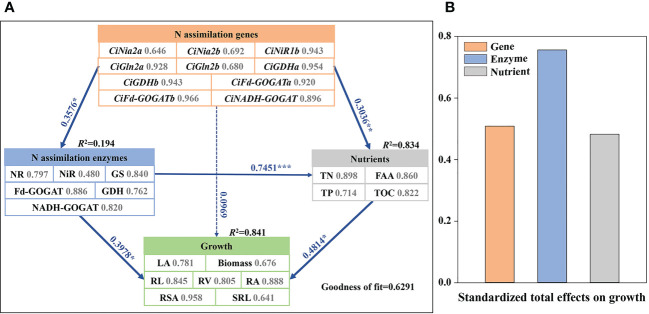
The partial least squares path models **(A)**. The partial least squares path models showing the effects of N assimilation genes, N assimilation enzymes, and nutrients on growth in pecan. The blue and red lines indicate positive and negative effects, respectively. Numbers adjacent to each arrow denote partial correlation coefficients (significance codes: ***p< 0.001, **p< 0.01, *p< 0.05). The number next to each indicator represents the contribution of each predictor variable to the latent variable. R^2^ values display the proportion of variance explained for each factor. **(B)**. The bar chart showing the standardized total effect of each factor on the growth composition in pecan. NR, Nitrate reductase; NiR, Nitrite reductase; GS, Glutamine synthetase; GOGAT, Glutamate synthase; GDH, Glutamate dehydrogenase; TN, Total nitrogen; TP, Total phosphorus; FAA, Free amino acid; TOC, Total organic carbon; LA, Leaf area; SRL, specific root length; RL, Root length; RSA, Root surface area; RV, Root volume; RA, Root activity.

The partial least squares path model (PLS-PM), which has been widely used to study complex multivariate relationships among variables (Latan and Noonan, 2017), was performed to infer potential direct and indirect effects of N assimilation genes, N assimilation enzymes, and nutrients factors on growth of pecan (**Figure 8**). The PLS-PM differs from the conventional covariance-based path analysis, and does not impose any distributional assumptions on the data which is usually difficult to meet. According to the results of PLS-PM analysis, plant nutrients had the main direct contribution to pecan growth, with TN, FAA, and TOC having the largest contribution to plant nutrients (contribution ratio > 0.8). N assimilation genes had a direct contribution to N assimilatory enzyme activity, with *CiNiR1b*, *CiGln2a*, *CiGDHa*, *CiGDHb*, *CiFd-GOGATa*, *CiFd-GOGATb*, and *CiNADH-GOGAT* contributing more to N assimilation genes (contribution ratio >0.8). In addition, N assimilating enzymes were positively regulating pecan growth and nutrients, with GS, Fd-GOGAT, and NADH-GOGAT contributing more to N assimilating enzymes (contribution ratio >0.8). The main effect analysis showed that N assimilating enzymes had the greatest total effect on pecan growth.

### 3.8 Comprehensive evaluation of N assimilation of pecan

**Figure 9:**
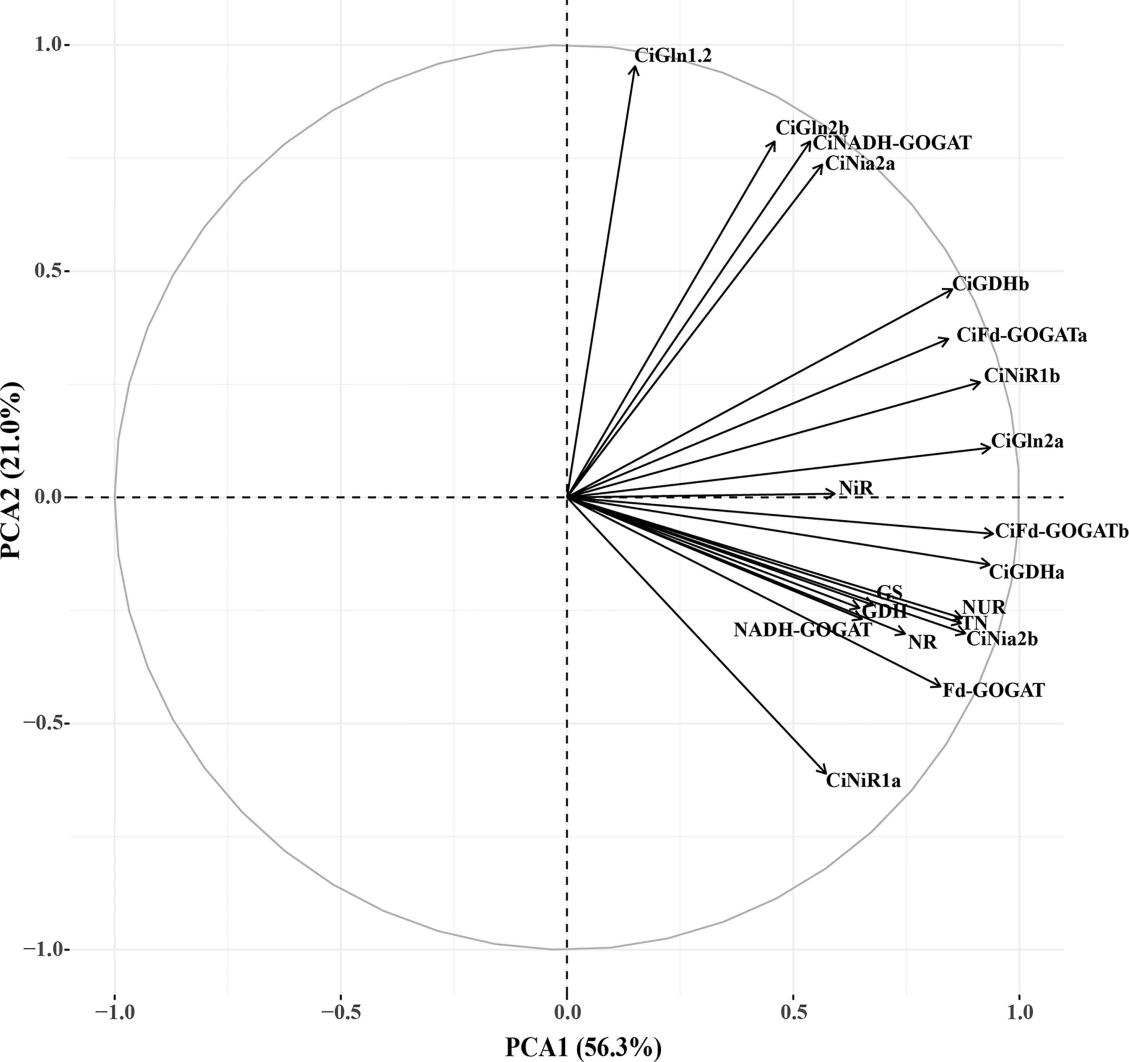
Factor loads of PCA. NUR, nitrogen uptake rate; NR, Nitrate reductase; NiR, Nitrite reductase; GS, Glutamine synthetase; GOGAT, Glutamate synthase; GDH, Glutamate dehydrogenase; TN, Total nitrogen.

In order to objectively evaluate the effects of the ${\text{NH}}_{4}^{\;+}$ : ${\text{NO}}_{3}^{\;-}$ treatments on the N assimilation, a principal component analysis was performed on 20 N assimilation traits, and the two principal components with the largest eigenvalues were obtained. The eigenvalues of the first and second principal components were 11.261 and 4.194, respectively. The cumulative contribution of the two principal components was 77.276% (**Table 3**), suggesting that the common factor can contain 77.276% of the original data information without losing variables. The factor loadings of the first and second principal components were performed on the X-axis and Y-axis, respectively (**Figure 9**). In the first principal component, the indexes with higher load (> 0.7) were TN, N uptake rate, *CiNia2b*, *CiNiR1b*, *CiGln2a*, *CiFd-GOGATa*, *CiFd-GOGATb*, *CiGDH1a*, *CiGDH1b*, NR, and Fd-GOGAT, indicating that the main factors determining the first principal component. In the second principal component, the indicators with larger load (> 0.7) were *CiNia2a*, *CiGln1.2*, *CiGln2b*, and *CiNADH-GOGAT*, which were the main factors determining the second principal component. We weighted the contribution of the principal components to calculate the combined scores under different ${\text{NH}}_{4}^{\;+}$ : ${\text{NO}}_{3}^{\;-}$ treatments, and then ranked (**Table 4**). The results showed that the comprehensive scores of the different treatments were T4 > T1 > T5 > T3 > T2 > CK. Except for T1 and T4 treatments, the scores of all other treatments were negative, indicating that T4 had a better promoting effect on the N assimilation of pecan seedlings than did the other treatments.

**Table 3:** The rate of eigenvalue, contribution, and cumulative contribution in principal components.

Principal Components	Eigenvalues	Contribution Rate/%	Cumulative Contribution Rate/%
1	11.261	56.306	56.306
2	4.194	20.970	77.276

**Table 4:** Scores of NH_4_^+^:NO_3_^–^ ratio treatments in the principal component and comprehensive evaluation.

Treatments	Z1	Z2	Comprehensive Score	Ranking
CK	-4.5075	0.3975	-2.4525	6
T1	1.8075	4.0125	1.8600	2
T2	-2.3125	-1.2875	-1.5700	5
T3	-0.6900	-1.7475	-0.7550	4
T4	5.8825	-1.5850	2.9800	1
T5	-0.1900	0.2050	-0.0600	3

## 4 Discussion

### 4.1 Effects of N forms on the growth and nutrient accumulation of pecan

The application of N fertilizer is an important way to improve the productivity of cash crops (Negrini et al., 2020). However, the effect of different forms of N fertilizer on plant growth is different (Nicodemus et al., 2008; Lang et al., 2018; Qin et al., 2020). We found that the ammonium-nitrate mixture promoted the growth and nutrient accumulation of pecan to different degree, Ammonia–Nitrate Mixture Dominated by ${\text{NH}}_{4}^{\;+}$ –N had the best effect (Chen et al., 2021).

We found that T4 and T5 treatments significantly increased aboveground biomass and RGR by further study. However, Kim et al. (2002) found that biomass of pecan decreased instead when ${\text{NH}}_{4}^{\;+}$ : ${\text{NO}}_{3}^{\;-}$ ratio was 75:25, which might be caused by the different levels of N addition, and the high ammonium concentration could lead to plant toxicity. Both ${\text{NH}}_{4}^{\;+}$ and ${\text{NO}}_{3}^{\;-}$ addition treatments increased but didn’t have significant differences in the underground biomass of pecan compared to the N deficiency treatment, and a significant difference existed in root length, root area, root volume, SRL, and root activity of pecan. This suggests that the different ${\text{NH}}_{4}^{\;+}$ : ${\text{NO}}_{3}^{\;-}$ ratio treatments may only have a unilateral promotion effect on the horizontal or vertical growth of pecan roots. It has been suggested that ${\text{NH}}_{4}^{\;+}$ promotes the emergence of lateral roots to establish a highly branched root system (Meier et al., 2020), which may be one of the reasons why the T4 and T5 treatments significantly increased the root area of pecan. The accumulation of FAAs is the result of biosynthesis and catabolism, and Yang et al. (2020) showed that both single addition of ${\text{NH}}_{4}^{\;+}$ and ammonia-nitrate mixture significantly increased the total FAAs in tea (*Camellia sinensis* L.). The results of our study were generally agreed with them, as the T3, T4, and T5 treatments all significantly increased the FAA concentrations of pecan. Previous studies have suggested that N and P have interaction in plants, and the supply of N usually increases the efficiency of P acquisition and use by plants, which was consistent with our findings (Güsewell, 2004). In general, ${\text{NH}}_{4}^{\;+}$ uptake by plants decreases rhizosphere pH, while weak acid conditions are more favorable for P absorption (Barrow, 2017), and our study also found that TP concentrations significantly increased under the condition of single addition of ${\text{NH}}_{4}^{\;+}$ . In addition, TK concentrations in roots increased significantly when ${\text{NH}}_{4}^{\;+}$ : ${\text{NO}}_{3}^{\;-}$ ratio was 50:50, which was consistent with the findings of Wang and Below (1998). The growth and development of plants are highly dependent on the interaction between C and N metabolism. Plants need to absorb a large amount of N into the photosynthesis mechanism, and also need a large amount of fixed C to provide C skeleton as a receptor in the assimilation process of inorganic N (Krapp and Traong, 2006). N can promote photosynthesis to produce more carbon substrates (Coruzzi and Bush, 2001). This study found that different forms of N addition increased the TOC concentration of plants, but with the decrease of ${\text{NO}}_{3}^{\;-}$ , the TOC concentration was higher, which may be due to the assimilation of ${\text{NO}}_{3}^{\;-}$ consumes more ATP and C skeleton (Nunes-Nesi et al., 2010). According to the results of PLS-PM analysis, TN and TOC were critical factors for plant growth, and T4 treatment significantly increased the FAA concentrations, TOC concentrations, TN concentrations, and NUE of pecan, indicating that ammonium-nitrate mixture dominated by ${\text{NH}}_{4}^{\;+}$ would be more beneficial to the growth of pecan, which was likewise confirmed by the results of principal component analysis.

### 4.2 Effects of N forms on the N uptake and assimilation of pecan

N assimilation of plants is the process by which they absorb ${\text{NH}}_{4}^{\;+}$ or ${\text{NO}}_{3}^{\;-}$ to synthesize nitrogenous organic compounds such as amino acids and proteins. The main enzymes involved in the assimilation of ${\text{NO}}_{3}^{\;-}$ are NR and NiR. In this study, both NR and NiR activities showed significantly greater in leaves than roots, which was attributed to the fact that most of the ${\text{NO}}_{3}^{\;-}$ absorbed by the root would be transported to the shoot and reduced in the mesophyll cells (Liu et al., 2022). Nitrate is both a nutrient and a signal to initiate various processes that trigger the induction of ${\text{NO}}_{3}^{\;-}$ assimilatory enzymes (Tischner and Kaiser, 2007). Therefore, NR and NiR activities in the leaves of pecan were significantly increased by a single addition of ${\text{NO}}_{3}^{\;-}$ . It has been suggested that ${\text{NH}}_{4}^{\;+}$ stimulates NR activity (Ivashikina and Sokolov, 1997), but a negative feedback effect of ${\text{NH}}_{4}^{\;+}$ on the ${\text{NO}}_{3}^{\;-}$ assimilation pathway has also been observed (Tischner, 2000), and the results of our study were the same as the former.

Regarding ${\text{NH}}_{4}^{\;+}$ assimilation of plant, the role of the GS/GOGAT cycle has been generally accepted (Zhao and Shi, 2006), while it still continues to be argued that the GDH shunt is also important and that it may be expected to play a deamination role in the tissue where amino acids are converted to transport compounds with low C/N ratios (Kant et al., 2007). Only a limited fraction of N is transferred in the organs after entering the plant, and a large fraction is released as NH_3_ and reassimilated *via* GS (Miflin and Habash, 2002). It is generally believed that ${\text{NH}}_{4}^{\;+}$ is mainly assimilated in roots (Ruan et al., 2016), and GS, GOGAT, and GDH activities all showed higher in leaves in this study, which may be due to differences in species and N supply levels (Feng et al., 2020), or it may be that ${\text{NH}}_{4}^{\;+}$ assimilation genes were mainly expressed in leaves of pecan. Low concentrations of external ${\text{NH}}_{4}^{\;+}$ and ${\text{NO}}_{3}^{\;-}$ had a positive effect on GS and GOGAT activity (Miflin and Habash, 2002; Balotf et al., 2016), which was consistent with the results of our study. Studies have shown that GDH activity increases under various stress conditions (Srivastava and Singh, 1987). GDH activity was significantly increased when ${\text{NH}}_{4}^{\;+}$ was added alone, which may be due to the role of GDH in relieving ammonium toxicity (Kant et al., 2007). Based on the results of the PLS-PM analysis, we suggested that pecan may enhance growth through increased N assimilation enzyme activity to accelerate ${\text{NH}}_{4}^{\;+}$ and. metabolism. The activities of all five N assimilating enzymes of pecan were significantly increased under T4 treatment, further indicating that ammonium-nitrate mixture dominated by ${\text{NH}}_{4}^{\;+}$ was more beneficial in promoting the growth of pecan.

### 4.3 Effects of N forms on the N assimilation-related genes expression of pecan

The PLS-PM results indicate that the N assimilate enzyme genes can influence nutrient uptake in pecan by regulating N assimilate enzyme activity, which ultimately affects growth of pecan, so the expression level of the N assimilate genes could indirectly reflect the growth status of pecan. In the assimilation of ${\text{NO}}_{3}^{\;-}$ , *Nia2* was responsible for 90% of the total NR activity in seedlings, whereas *Nia1* accounts for the remaining 10% (Yu et al., 1998). Previous studies suggested that ${\text{NO}}_{3}^{\;-}$ is a signal for ${\text{NO}}_{3}^{\;-}$ assimilation genes (Wang et al., 2004), and it can up-regulate the expression of *Nia* and *NiR* genes (Loppes et al., 1999; Konishi and Yanagisawa, 2010), which was consistent with the results of our study, where the expression levels of pecan *CiNia2a*, *CiNia2b*, and *CiNiR1b* were significantly up-regulated under single application of ${\text{NO}}_{3}^{\;-}$ . Loppes et al. (1999) also found that ${\text{NH}}_{4}^{\;+}$ down-regulated the expression level of *Nia* genes, but Kim et al. (2016) suggested that ${\text{NH}}_{4}^{\;+}$ up-regulated the expression level of *nia2*, and our study were consistent with the former, with *CiNia2a*, *CiNia2b*, and *CiNiR1b* expression levels significantly down-regulated under single application of ${\text{NH}}_{4}^{\;+}$ . The response of critical ${\text{NO}}_{3}^{\;-}$ ${\text{NO}}_{3}^{\;-}$ assimilation genes to ${\text{NO}}_{3}^{\;-}$ may be independent of ${\text{NO}}_{3}^{\;-}$ reduction (Wang et al., 2004), and our study found no significant correlation between *CiNia2a* and NR, perhaps as *Nia2* gene was also involved in the regulation of chlorate resistance (Wilkinson and Crawford, 1991). Loqué et al. (2003) also showed that the *AtNIA2* gene was overexpressed in the roots of NR-deficient mutants.

In the *Arabidopsis* genome, one *Gln2* gene and five *Gln1* genes are encoded (Taira et al., 2004). *Gln1;2* is essential for N assimilation and ammonia detoxification, and *gln1;2* mutants exhibit lower GS activity (Lothier et al., 2011). Under high ${\text{NH}}_{4}^{\;+}$ treatment, the expression level of *AtGln1;2* in shoots was up-regulated, the expression level of *AtGln1;1*, *AtGln1;4*, and *AtGln1;5* was down-regulated, and the expression level of *AtGln1;3* was unchanged (Guan et al., 2016). The expression pattern of *CiGln1.2* was similar to that of *AtGln1;2*, and the expression level of *CiGln1.2* in leaves was significantly up-regulated by single treatment with ${\text{NO}}_{3}^{\;-}$ , indicating that *CiGln1.2* also has an important role in ${\text{NH}}_{4}^{\;+}$ assimilation and ammonium detoxification in pecan leaves. Wang et al. (2021) found that *Gln2* was up-regulated in shoots when ${\text{NH}}_{4}^{\;+}$ was the only N source, which was consistent with the results of our study. However, recent studies have shown that *gln2* mutants do not exhibit an abnormal phenotype and that the isozyme encoded by the *GS1* genes, may mask the *gln2* mutation in *Arabidopsis* shoots (Lee et al., 2022). In case of ${\text{NH}}_{4}^{\;+}$ assimilation genes, ${\text{NO}}_{3}^{\;-}$ is also a direct signal (Wang et al., 2004), and *CiGlns* were significantly up-regulated in leaves of pecan under T1 treatment. Additionally, *CiGln2s* were highly expressed in the leaves but very low levels in the roots, which agreed with the study of Guan et al. (2016).

GOGAT is mostly found in the plasmid, and Fd-GOGAT accounts for 96% of the total GOGAT activity (Bi et al., 2017). Studies have shown that the *Fd-GOGAT* gene acts primarily in leaves, while the *NADH-GOGAT* gene contributes more to root ${\text{NH}}_{4}^{\;+}$ assimilation (Konishi et al., 2014), which was also confirmed in poplar (*Populus* L.) (Cao et al., 2023). However, this was not entirely accordance with the results of our study, where *CiFd-GOGATs* were expressed at higher levels in leaves except for CK treatment, whereas *CiNADH-GOGAT* was expressed at higher levels in roots under CK, T2, and T3 treatments, which may be due to differences in species and N levels. (Balotf et al., 2016) found that high concentrations of ${\text{NH}}_{4}^{\;+}$ and ${\text{NO}}_{3}^{\;-}$ could increase the expression level of GOGAT genes, sand the transcript level of GOGAT genes decreased under N-deficient conditions. This was the same with our findings that the expression level of pecan GOGAT gene was significantly up-regulated in leaves under the addition of ${\text{NH}}_{4}^{\;+}$ or ${\text{NO}}_{3}^{\;-}$ alone, while *CiFd-GOGATb* and *CiNADH-GOGAT* were significantly down-regulated under CK treatment except for *CiFd-GOGATa*. Importantly, T4 treatment significantly up-regulated the pecan GOGAT gene, indicating that the addition of ammonium-nitrate mixtures dominated by ${\text{NH}}_{4}^{\;+}$ was more favorable for pecan ${\text{NH}}_{4}^{\;+}$ assimilation.

In the model plant *Arabidopsis*, besides the GS/GOGAT cycle, GDH has a complementary role in N assimilation under specific physiological conditions of high ${\text{NH}}_{4}^{\;+}$ concentrations (Glevarec et al., 2004). In this study, we found that not only T5 treatment up-regulated the expression level of *CiGDHs* in pecan leaves, but also T1, T3, and T4 treatments, suggesting that the expression level of *CiGDHs* can also be regulated by ${\text{NO}}_{3}^{\;-}$ . Although the expression patterns of *CiGDHa* and *CiGDHb* were almost identical under different N form treatments, GDH was highly significantly positively correlated with *CiGDHa*, implying that *CiGDHa* may play a more important role in pecan ${\text{NH}}_{4}^{\;+}$ assimilation.

## 5 Conclusions

We analyzed the effects of different N form treatments on the growth, nutrient uptake, and N assimilation of pecan, and found that ${\text{NH}}_{4}^{\;+}$ was more beneficial to promote the growth, N uptake and assimilation of pecan, and the ammonium-nitrate mixture dominated by ${\text{NH}}_{4}^{\;+}$ had the best effect. The results of PLS-PM analysis also indicated that N uptake and assimilation would directly affect the growth of pecan. Furthermore, correlation analysis showed that N assimilate enzyme activity did not necessarily correlate positively with N assimilate gene expression level, partly because N assimilate enzymes are not only regulated by N assimilate genes, and partly because some N assimilate genes have other functions than regulating N assimilate enzymes. Therefore, we believe that the N assimilation ability of plants should not be judged only by N assimilation genes in future studies, which should be confirmed by a comprehensive analysis of N concentration, N assimilation enzymes, and related genes. The study provides a basis for further identification of the functions of N assimilation genes in the N assimilation process of pecan, and also provides a theoretical support for improving the yield of pecan by improving NUE, and promoting the scale development of pecan.

## Data availability statement

The original contributions presented in the study are included in the article/**Supplementary Material**. Further inquiries can be directed to the corresponding author.

## Author contributions

FP conceived and designed the study. MC collected experimental data, analyzed, and wrote the manuscript. MC and JL participated in the collection of samples. MC, ZQ, and JX performed the experiments. PT and KZ provided help in data analysis and in improving the manuscript. All authors contributed to the article and approved the submitted version.

## Supplementary material

Supplementary Figure 1 Melt curves of 12 selected genes.
