# Supplementary figures and images for: Ammonium-nitrate mixtures dominated by NH4 +-N promote the growth of pecan (Carya illinoinensis) through enhanced N uptake and assimilation
Source: Front Plant Sci. 2023 May 29;14:1186818. doi: 10.3389/fpls.2023.1186818 (PMC10258329; doi:10.3389/fpls.2023.1186818)

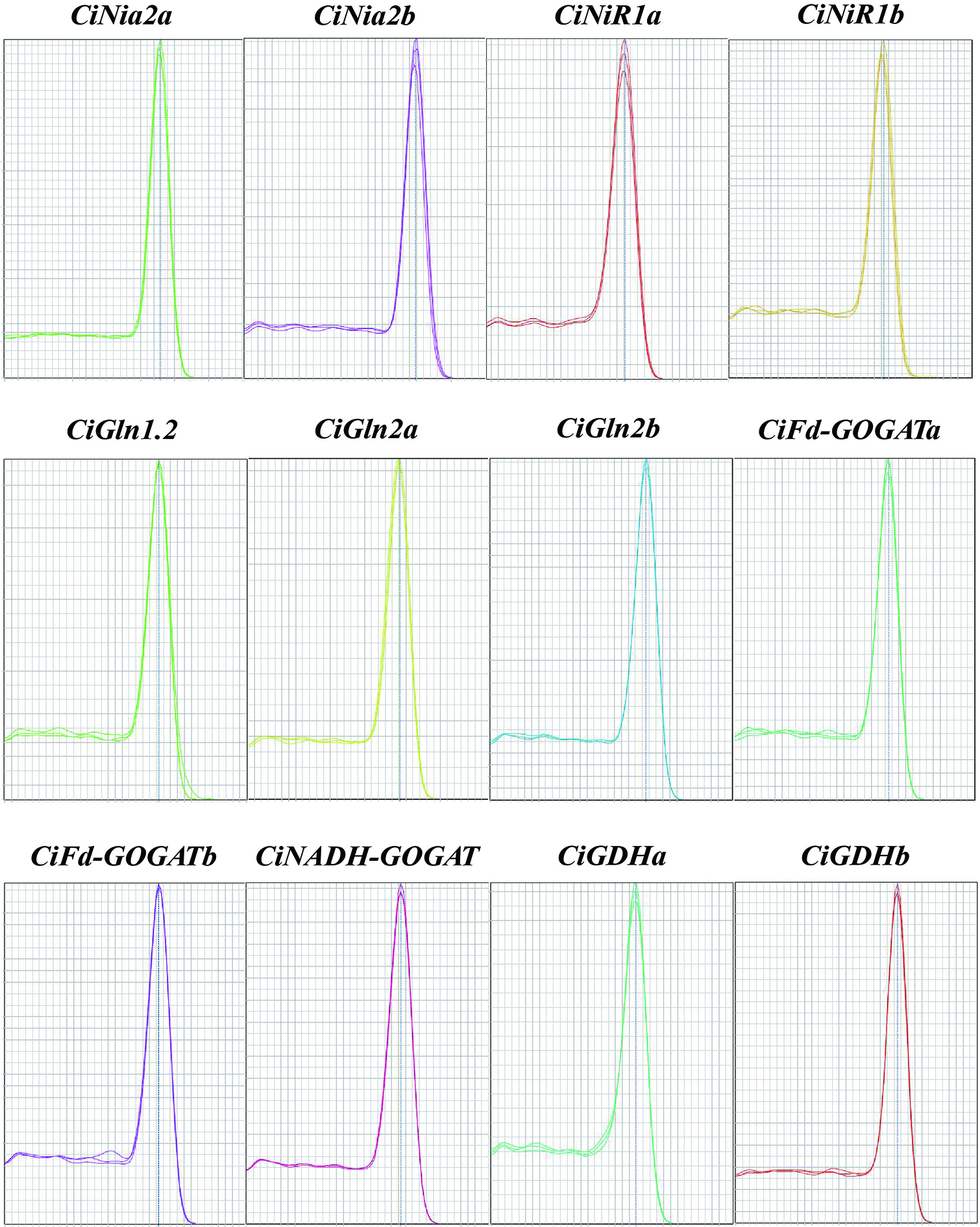

Supplement: Supplementary Figure 1 — Melt curves of 12 selected genes. [file Image_1.jpeg]
